# Artificial intelligence for global health: cautious optimism with safeguards

**DOI:** 10.2471/BLT.23.290215

**Published:** 2023-06-01

**Authors:** Sameer Pujari, Andreas Reis, Yu Zhao, Shada Alsalamah, Fatima Serhan, John C Reeder, Alain B Labrique

**Affiliations:** a Digital Health and Innovation Department, World Health Organization, Avenue Appia 20, 1211 Geneva 27, Switzerland.; b Research for Health Department, World Health Organization, Geneva, Switzerland.; c Office of the Chief Scientist, World Health Organization, Geneva, Switzerland.

The United Nations Secretary-General has stated that the safe deployment of new technologies, including artificial intelligence, can help the world to achieve the sustainable development goals.^1^

The rapid diffusion and growing number of applications of artificial intelligence large language models has generated excitement and public discourse around their potential to improve human health. However, this enthusiasm has been accompanied by concerns that such content-generative systems may be biased, produce misleading or inaccurate information, and could relinquish data privacy and ownership controls to technology firms looking to commercialize large language models and commodify data.^2^ Some have questioned whether commercial pressures have led to public releases of these technologies without adequate ascertainment of their safety and performance.^3^

Large language models generate responses that can appear authoritative and plausible to an end-user; however, without adequate controls in place, the veracity and accuracy of responses may be extremely poor.^4^ These models may be trained on data for which explicit consent may not have been provided, and they may not protect sensitive data (including health data) that users voluntarily feed into the artificial intelligence-based tool. Large language models, usually trained on large amounts of raw data, may encode biases in the data that can undermine inclusiveness, equality and equity.^5^ Furthermore, building such large data models has an environmental (mostly in carbon dioxide emissions) and financial impact that is often overlooked in costing analyses.^6^

Artificial intelligence tools are increasingly being applied to public health priorities,^7^ and have the potential to assist with pattern recognition and classification problems in medicine – for example, early detection of disease, diagnosis and medical decision-making.^8^^,^^9^ The increase in sophistication of artificial intelligence systems is now marked in days and weeks, as opposed to months and years. This speed outpaces the regulatory and review capacity of most agencies charged with protecting public health and providing oversight of technologies applied to health and well-being.

For artificial intelligence to have a beneficial impact on global health, especially in low- and middle-income countries, ethical considerations, regulations, standards and governance mechanisms must be placed at the centre of the design, development and deployment of artificial intelligence-based systems. The proliferation of artificial intelligence for health must take place with oversight by governments and their appropriate regulatory agencies. Acknowledging the enthusiasm sparked by emerging positive evidence of high-performing artificial intelligence systems in disease diagnostics, integrating complex patient histories to enhance clinical decision support, or health system quality improvement modelling, requisite caution is warranted given the precipitous pace of progress in recent months. Improved transparency and fail-safes are needed to ensure safety, consistency and quality in artificial intelligence systems for health, while promoting trust. As the amount of textual, audio or video content generated by or with the help of artificial intelligence grows, consumers of health information may find it difficult to assess content validity and reliability. Clear acknowledgement of the extent of human expert oversight or other quality control measures taken may be warranted and helpful. 

The World Health Organization (WHO) is responding to this fast-paced change through strategic interventions in line with the *Global strategy on digital health*.^10^ WHO is providing guidance to Member States to develop an appropriate regulatory environment that can oversee the selection, evaluation and eventual deployment of such technologies. To this end, WHO has published guidance on *Ethics and governance of artificial intelligence for health*,^11^ and has convened an expert group to develop additional guidance.

WHO encourages policy-makers to prioritize the implementation of standards and evaluative frameworks that promote the responsible development and application of such technologies, working closely with technical experts, civil society and the private sector to identify risks, and develop mitigation strategies that preserve public health and foster trust. We should also acknowledge the sensationalism of the news cycle and social media exaggerations, and examine emerging capabilities and risks dispassionately and empirically. Companies developing health-related artificial intelligence should be encouraged to act as responsible stewards of public health by prioritizing the well-being and safety of individuals above commercial interests, implementing WHO-recommended guidance and best practices even in poorly regulated environments.

In 2018, WHO and the International Telecommunications Union (ITU) established the WHO-ITU Focus group on artificial intelligence for health. This collaboration convened more than 100 stakeholders to develop a benchmarking framework to guide the design, development, regulation and deployment of these tools that bring health benefits to everyone, everywhere. A multiagency global initiative on artificial intelligence for health is warranted to improve coordination, leverage collective and individual agency capacity, and ensure that the evolution of artificial intelligence steers away from a dystopian future towards one that is safe, secure, trustworthy and equitable.

## References

[1] Report of the Secretary-General on SDG progress. New York City: United Nations; 2019. Available from: https://sustainabledevelopment.un.org/content/documents/24978Report_of_the_SG_on_SDG_Progress_2019.pdf [cited 2020 Nov 8].

[2] Pan X, Zhang M, Ji S, Yang M. Privacy risks of general-purpose language models. In: 2020 IEEE Symposium on Security and Privacy (SP); 2020 May 18–20; San Francisco, United States of America. New York: IEEE Computer Society’s Technical Community on Security and Privacy; 2020. 10.1109/SP40000.2020.00095 10.1109/SP40000.2020.00095

[3] Harrer S. Attention is not all you need: the complicated case of ethically using large language models in healthcare and medicine. EBioMedicine. 2023 Apr;90:104512. 10.1016/j.ebiom.2023.104512 36924620 PMC10025985

[4] Samo G, Bonan C, Si F. Health-related content in transformer-based deep neural network language models: exploring cross-linguistic syntactic bias. Stud Health Technol Inform. 2022 Jun 29;295:221–5. 10.3233/SHTI220702 35773848

[5] Sheng E, Chang K-W, Natarajan P, Peng N. The woman worked as a babysitter: on biases in language generation. In: Proceedings of the 2019 Conference on Empirical Methods in Natural Language Processing and the 9th International Joint Conference on Natural Language Processing (EMNLP-IJCNLP); 2019 Nov 3–7; Hong Kong, China. Hong-Kong: Association for Computational Linguistics; 2019.

[6] Bender EM, Gebru T, McMillan-Major A, Shmitchell S. On the dangers of stochastic parrots: can language models be too big? In: Proceedings of the 2021 ACM Conference on Fairness, Accountability, and Transparency (FAccT '21); 2021 Mar 3–10; Virtual Event, Canada. New York: Association for Computing Machinery; 2021. Available from: https://dl.acm.org/doi/pdf/10.1145/3442188.3445922 [cited 2023 May 10]. 10.1145/3593013.3594102 PMC10661580 37990734

[7] Amisha MP, Malik P, Pathania M, Rathaur VK. Overview of artificial intelligence in medicine. J Family Med Prim Care. 2019 Jul;8(7):2328–31. 10.4103/jfmpc.jfmpc_440_19 31463251 PMC6691444

[8] Topol EJ. High-performance medicine: the convergence of human and artificial intelligence. Nat Med. 2019 Jan;25(1):44–56. 10.1038/s41591-018-0300-7 30617339

[9] Wahl B, Cossy-Gantner A, Germann S, Schwalbe NR. Artificial intelligence (AI) and global health: how can AI contribute to health in resource-poor settings? BMJ Glob Health. 2018 Aug 29;3(4):e000798. 10.1136/bmjgh-2018-000798 30233828 PMC6135465

[10] Global strategy on digital health 2020–2025. Geneva: World Health Organization; 2021. Available from: https://apps.who.int/iris/handle/10665/344249 [cited 2023 May 10].

[11] Ethics and governance of artificial intelligence for health: WHO guidance. Geneva: World Health Organization; 2021. Available from: https://www.who.int/publications-detail-redirect/9789240029200 [cited 2023 May 10].

